# Occupational exposure to hepatitis C virus infection and associated factors among healthcare workers in Fattouma Bourguiba University Hospital, Tunisia

**DOI:** 10.1371/journal.pone.0274609

**Published:** 2022-09-13

**Authors:** Meriem Kacem, Wafa Dhouib, Cyrine Bennasrallah, Imen Zemni, Hela Abroug, Manel Ben Fredj, Arwa Guedich, Leila Safer, Nissaf Ben Alaya, Maha Mastouri, Ines Bouanene, Asma Sriha Belguith

**Affiliations:** 1 Department of Preventive Medicine, University of Monastir, Monastir, Tunisia; 2 Department of Gastroenterology, University of Monastir, Monastir, Tunisia; 3 National Observatory of New and Emerging Diseases, University of Manar, Manar, Tunisia; 4 Laboratory of Microbiology, University of Monastir, Monastir, Tunisia; Centre de Recherche en Cancerologie de Lyon, FRANCE

## Abstract

**Background:**

Healthcare workers (HCWs) are at high risk of hepatitis C virus (HCV) infection. Indeed, they are exposed to blood and body fluid which put them at an important risk of transmission of various blood borne pathogens including HCV. The goal of this study was to determine the magnitude of occupational exposure to hepatitis C virus infection as well as the factors associated to this exposure among HCWs at a Tunisian University Hospital in 2017.

**Methods:**

A hospital-based cross-sectional study was carried out at Fattouma Bourguiba University Hospital in Monastir Governorate (Tunisia) from 01 June 2017 to 31 August 2017. Data were collected using an anonymous questionnaire. To determine factors associated with occupational exposure to hepatitis C virus infection, we performed multivariate analysis.

**Results:**

Among the 1493 included participants, more than half (56.7) had at least one exposure to blood or body fluid. A history of needle stick injury was reported by 48.3% of the respondents. Exposure to blood or body fluid splash into the face was announced by 32.1% HCWs. Doctors had the highest risk of exposure (AOR = 12.425; 95% CI: [05.310–29.075]). Participants working at surgical departments were the most exposed comparing to workers at others departments (AOR = 7.440; 95% CI: [4.461–12.408]). Two exposed female HCWs were tested positive corresponding to a HCV infection prevalence of 0.13% (95% IC: [0.11–0.16%]).

**Conclusion:**

Occupational exposure to hepatitis C virus infection was high at the university hospital of Monastir. Despite the low magnitude of HCV infection, preventive actions should be taken to promote the safety of health care personnel.

## Introduction

Globally, Viral hepatitis C (HCV) infection is a public health problem [1]. Its prognosis may be severe and can be responsible of an important burden related to work disability or to death by cirrhosis and liver cancer [2,3]. Among the chronically infected HCV patients, 80% are asymptomatic which are silent carriers and cause of high transmission rate [4]. HCV transmission occurs mainly by parenteral exposure to infectious blood or body fluids through injuries to the skin or mucous membranes [4,5].

Healthcare workers (HCWs) have a high risk of HCV infection. Indeed, health professionals have the most dangerous biohazard comparing to others professionals [6]. HCWs are particularly exposed to various blood borne pathogens primarily hepatitis B virus (HBV), HCV and HIV [7]. Repeating the performance of exposure prone procedures is an important risk for health professional. Sharp injuries to HCWs feature among the main occupational accidents in healthcare, however, many of them are still unreported [8–10]. Many factors are associated to the risk of seroconversion after an occupational blood exposure such as the injury type, the virus load in the index patient, the amount of infectious material transmitted and genetic characteristics of the injured worker [8]. Therefore, the prevalence of HCV infection among exposed HCWs is variable. In previous studies, it is estimated at 0%–17% [8,11–13].

Infected HCWs can transmit HCV virus to their patients [14]. Therefore, the prevention of HCV transmission to health care givers and treating them if infected, is very important to break the chain of HCV transmission and to alleviate the burden of this infection. The primary prevention of HCV is only possible by avoiding exposures since vaccination does not exist [15]. Treating HCV infection become possible due to the advent of direct-acting antivirals (DAAs) [16].

Describing the magnitude of occupational exposure to HCV infection and its associated factors is necessary for the safety of both HCWs and patients. In Tunisia, health professional exposure to HCV still poorly explored. Tunisian publications on HCV infection among HCWs are rare [17].

We aimed to determine the magnitude of occupational exposure to hepatitis C infection and its associated factors among HCWs at the University Hospital of Fattouma Bourguiba of Monastir (Tunisia) in 2017.

## Methods

### Setting and design

We carried out a hospital based cross-sectional study at Fattouma Bourguiba University hospital (FBUH) in Monastir governorate from 01 June 2017 to 31 August 2017. Monastir is a city in east central of Tunisia. FBUH includes 10 surgical departments, 23 medical departments, six laboratories, administrative and pharmacy wards with a total of 2020 healthcare workers (HCWs).

### Population

The study population included health workers from all departments and categories (physicians, dentists, anesthetists, midwives, nurses, laboratory personnel/technicians, housekeeping, administrative, security and pharmacy staff) at FBUH who accepted to participate to the study. Considering a prevalence of HCV infection of 1%, alpha error of 5% and a marginal error of 0.5%, the required sample size was 1521 HCWs and we added 20% (in order to replace the number of non-responders), so the estimated sample size was of 1825 participants.

### Variables

Occupational exposure: defined as an exposure to blood or body fluid all types combined: exposure to sharp injury or/and blood or body fluid splash into eyes, face or injured skin.

### Measurements

Five milliliters of venous blood were collected from each participant. The samples were transported to the FBUH microbiology Laboratory for analysis. A 3rd generation ELISA (Enzyme-Linked Immunosorbent Assay) test (Abbott®) was performed as a screening test for all subjects included in the survey. A result was considered positive for anti-HCV antibodies if the serum tested had an absorbance ratio greater than one. For Samples found positive or doubtful at initial screening, a second test, was performed on a different sample serving as a control using a 3rd generation immunoblot test. The positivity of a result is defined by the identification of at least two viral proteins. A search for viral RNA for HCV using a qualitative PCR technique was performed for subjects diagnosed positive for the two anti-HCV antibody tests.

### Data collection

An anonymous structured French version questionnaire was used to collect data on demographic characteristics (age, sex, occupation, department, length of employment), on the potential presence of HCV infection risk factors, on the number, place and circumstances of accidents during working hours, as well as on the measures taken after an accident. Data collection from HCWs was facilitated by medical interns using a confidential interview.

### Data analysis

Data were collected and analyzed using SPSS version 20 software. To describe the study population quantitative variables were expressed by means and standard deviations(SD) or medians and interquartile range (IQR) and qualitative ones were by percentages.

We explored factors associated with occupational exposure to hepatitis C virus infection by a bivariate statistical analysis: Chi2 test, or the Fisher test for comparison of frequencies and student t test for comparison of means and a multivariate statistical analysis by logistic regression including all variables with a p-value < 0.25 in the bivariate analysis.

### Ethical considerations

This study was carried out under Good Clinical Practice conditions using ethical standards collections. The medical and research ethics committee of the University Hospital of Farhat Hached of Sousse approved the protocol. We submitted a letter of permission to FBUH administration body and to each head of different hospital departments. We explained the objective of the study to each participant. Every entrant was informed that the survey was composed by blood sample collection for HCV screening and interviews. We informed them that the participation to the study is voluntary. Moreover, participants could withdraw from the study during the interview. We obtained a written informed consent for participation from each volunteer. We ensured participants that their test results were kept confidential due to a unique code specific to each study participant. Laboratory personnel had only access to the unique codes written on test tubes. Participants’ identifiers (name and phone number) were written in a separate file. Only the research team members had access to this file. All participants were informed of the result in an individual letter containing the test result and preventive recommendations. Participants with positive result were linked to gastroenterology service for counseling and care.

## Results

### Socio-demographic characteristics

A total of 1493 HCWs of FBUH were tested through this study corresponding to a total response rate of 81%. Their median age was of 36 years (IQR: 31–46 years). Women represented 73% of the study population corresponding to a sex ratio of 0.36. The majority of the responders were nurses (34.6%). Participants had a median work experience of seven years (IQR: 3–16 years). The study covered responders mainly from medical departments (40.5%) and surgery ones (39%). Almost half (50.4%) of the participants were resident in Monastir city. Among included HCWs, 480 (33.4%) had previous HCV antibodies test performed mainly in the context of pregnancy or prenuptial assessment (17%). All had a negative result. See details in Table 1.

**Table 1:** Description of sociodemographic characteristics of HCWs of FBUH (Monastir 2017).

Characteristics	N	%
**Sex**		
Man	384	26.8
Woman	1048	73.2
**Age**		
≤ 30	345	24.6
31–35	309	22.1
36–40	237	16.9
>40	510	36.4
**Work experience (years)**		
≤5	478	40.1
6–10	282	23.7
11–15	118	9.9
>15	314	26.3
**Profession**		
Physician	308	21.6
Nurses	493	34.6
Technician	314	22.0
Housekeeper	214	15.0
Administrative security and pharmacy staff	96	6.8
**Department**		
Medical	583	40.5
Surgical	561	39.0
Laboratory	141	9.8
Others	153	10.7

### Risky behavior and history of medical procedures

The majority (88.2%) of the responders did not have risky behaviors. For medical procedures, 38.8% of the participants reported a history of having surgical intervention and 34.8% of women had at least one caesarean operation. Twenty-one (1.4%) participants lived with household members infected with HCV (Table 2).

**Table 2:** Distribution of risky behaviors and history of medical procedures among HCWs (N = 1493).

Risk factor	N	%
**Behavioral**		
No behavioral risk	1318	88.2
Alcohol drinking	70	4.8
IV drug use	15	1
Multiple sexual partner/s	73	5.7
Practicing tattoos	36	2.5
Practicing piercing	65	4.6
Household member with HCV	21	1.4
**Medical procedures**		
Care outside Tunisia	153	10.8
Transfusion before 1987	51	3.5
Antecedents of surgical intervention	563	38.8
Antecedents of caesarean*	295	34.8
Antecedents of curettage*	283	33.7
Organ transplant	8	0.5
Acupuncture	23	1.6
Mesotherapy	12	0.8
Varicose sclerosis	12	0.8

*ratio per woman.

### Occupational exposure to hepatitis C virus infection

**Fig 1:**
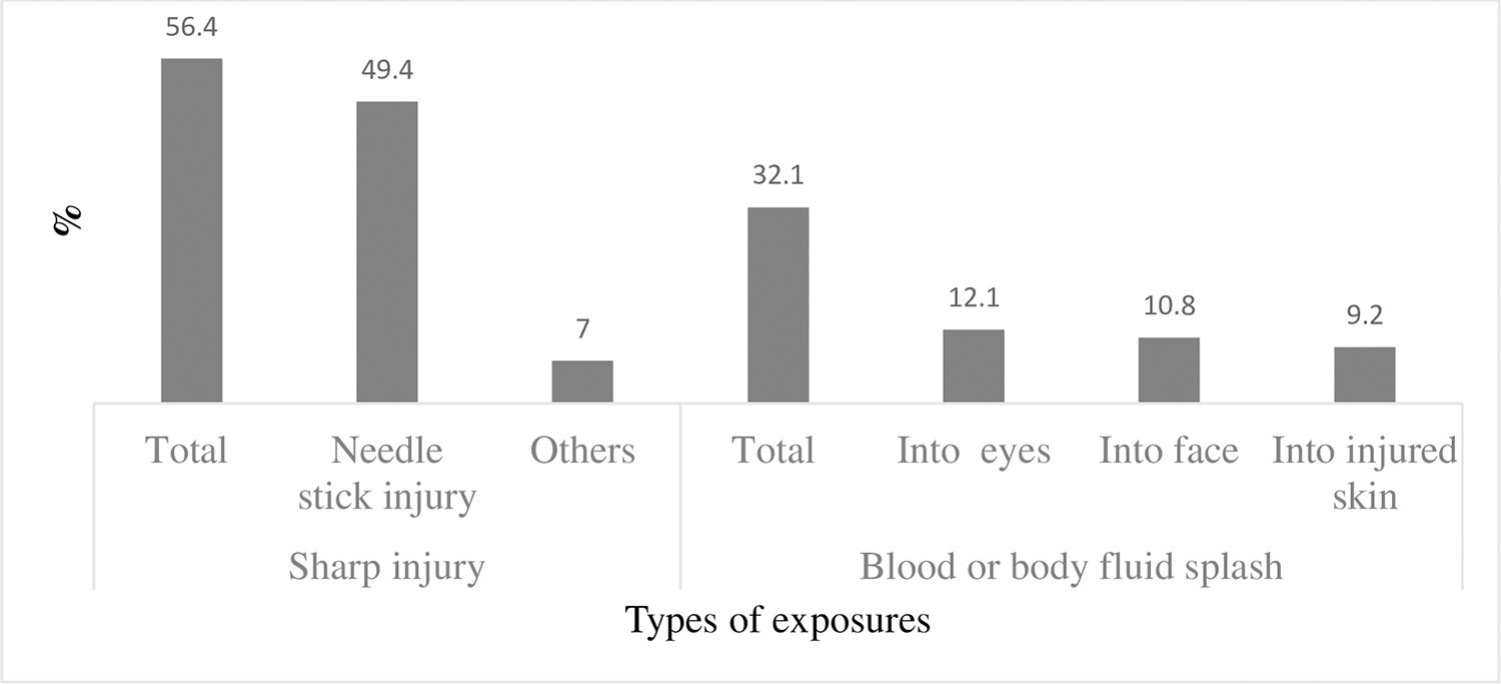
Types of occupational exposure to hepatitis C virus infection among health care workers.

More than half (56.7%, n = 804) of the interviewed HCWs, were exposed at least once to blood or body fluid. Twice or more OBE was reported among 35.4% (n = 502) of the participants. History of needlestick injury was reported by 48.3% (n = 701) of the respondents. For 32.1% (n = 455) of the study population we noted a history of blood or body fluid splash into the eyes and/or face and/or injured skin (Fig 1).

#### Magnitude of hepatitis C infection

Among 1493 HCWs tested for anti-HCV antibody, only two were positive corresponding to a prevalence of 0.13% (IC95%:0.11–0.15). Among exposed HCW, this prevalence was of 0.25% (IC%:0.22–0.28). The two were females. One was a nurse in a surgical department and had a history of sharp injury more than once (one exposure by scalpel). She reported having transfusion before 1987 and a history of surgical intervention and endoscopy. The second participant tested positive was a housekeeper and was exposed to a needle stick injury and had a history of scarification. Both had a good practice of standard precautions. For the two cases source patient was not identified and serological follow-up was not done after the accident. Both were addressed to the gastroenterology department for care. Viral RNA for HCV was tested negative for the housekeeper and she was considered as spontaneously recovered. The nurse had a positive viral RNA for HCV and she was linked to care.

#### Factors associated with occupational exposure to hepatitis C virus infection

The univariate analysis demonstrated that occupation and work place were significantly associated with occupational exposure of the participants to blood or body fluid.

Also in the multivariate analysis occupation and work place were independently associated with occupational exposure. Doctors were the most exposed to hepatitis C virus infection than other HCWs (AOR = 12.425; 95% CI: 05.310–29.075). Participants working at surgical departments had the highest risk of exposure comparing to those at others departments (AOR = 7.440; 95% CI: 4.461–12.408) (Table 3).

**Table 3:** Factors associated with occupational exposure of HCWs of FBUH to hepatitis C virus (Monastir, Tunisia 2017).

	Total*	OE:n	%	X^2**^	p-value	AOR**	95%CI**	p-value
**Total**	**1417**	**804**	**56.7**					
**Sex**	**1368**	786	57.5	3.397	0.060			
Men	360	192	53.3					
Women	1008	594	58.9					
**Work experience (years)**	**1140**	661	58	1.001	0.801			
< = 5 ans	460	272	59.1					
6–10 ans	268	151	56.3					
11–15 ans	113	63	55.8					
>15	299	170	56.9					
**Occupation**	**1362**	777	57.6	120.890	**<0.0001**			**<0.0001**
Physician	294	201	68.4			**12.425**	5.310–29.075	
Nurses	481	315	65.5			10.142	4.395–23.406	
Technician	297	146	49.2			6.345	2.722–14.788	
Housekeeper	205	107	52.2			6.597	2.792–15.587	
Administrative and pharmacy staff	85	8	9.4			1		
**Work place**	**1373**	791	57.6	144.603	**<0.0001**			**<0.0001**
Medical	561	328	58.5			3.925	2.355–6.542	
Surgical	538	381	70.8			**7.440**	4.461–12.408	
Laboratory	136	59	43.4			2.637	1.453–4.786	
Others	138	23	16.7			1		
**Age**	**1338**	768	57.4	1.864	0.601			
< = 30	330	199	60.3					
30–35	298	164	55					
36–40	227	130	57.3					
>40	483	275	56.9					

* Missing data were excluded

** X^2^: chi-square value, AOR: Adjusted Odds Ratio, 95% CI: Adjusted 95% Confidence Interval.

## Discussion

This study found an occupational blood or body fluid exposure (OBE) prevalence rate of 56.7% in a Tunisian university hospital. Doctors and participants working at surgical departments were more exposed than others categories. Of the 1493 HCWs tested, only two exposed women were tested positive.

More than half of the participants experienced at least one OBE. The prevalence of OBE was lower as that reported in a study from Morocco (64.38%) [18]. Another study from Bosnia and Herzegovina showed that 70% of HCWs reported at least one type of exposure [19]. In this study, almost half of the responders had a history of needle stick injury (NSI). Exposure to body fluids is another source for HCWs to contract blood-borne infection from workplace. Our survey showed that 32.1% of them had a history of blood or body fluid splash into the face. Alemayehu and colleague in Ethiopia have shown that 26.5% of service providers got sharp injury and 36.1% had exposure to blood and body fluids [20]. A study from an Algerian university hospital showed that NSI represented 81% of total exposures [21]. In Australia a study conducted revealed that 42% of HCWs had body fluid exposures during one year [22].

Participants working at surgical departments were more exposed than others. This is in accordance with the literature. Indeed, several studies have shown that there is higher incidence of accidents in operating theaters than in other wards [7,19,23]. Moreover, in the present study being Doctor was an independent risk to OBE. However, in others studies doctors were significantly less likely to report exposure incidents than nurses and support staff [7,19]. In a Korean survey, housekeeping showed the highest OBE rate [24]. Another study carried out at a tertiary care hospital in Mumbai (India) nurses were the most exposed to OBE whereas being nurse was a protective factor to OBE according to Alemayehu and colleague [20].

In our study sex was not a risk factor for exposure. In the literature results are contradictory, indeed being male decrease the risk of OBE in a study done in Eastern Ethiopia however another study in Jimma University Medical Center (Addis Ababa) showed the opposite [13,20].

The prevalence of hepatitis C infection among HCW tested in the present survey was 0.13% (IC95%:0.11–0.16%). This was comparable to the prevalence of hepatitis C infection in the general population at Monastir governorate (0.20%) [25]. Other studies also showed that the prevalence of HCV among HCW was comparable or lower than the general population [12,14].

The prevalence of HCV infection in this study was similar to the 0% prevalence reported among healthcare workers of the Najran region.Southwestern Saudi Arabia and those of a Damascus Hospital [12,26]. Other results from studies on university hospitals were close to our findings [13,14]. However, a systematic review by Westermann et al. concluded that HCWs in Europe, USA, Africa, Middle East and South Asia were most exposed than the general population especially those working in areas with high exposure to blood contact (e.g. dialysis, blood banks, etc.) but in Japan there was no difference detected in the risk of HCV between HCWs and general population [8].

In our study the seropositive HCWs were two women exposed to occupational blood exposures. Risk factors related to HCV infection among health staff were not explored in the present work. In the literature the risk of HCV transmission increase with several factors such as the deep of injuries and procedures involving hollow-bore needle placement in the source patient’s vein or artery, viral load of the source patients, professional experience (etc.) [6,27].

This study had several limitations. First, it was limited to HCWs in a tertiary hospital; thus the results may not be generalizable to other settings. Nonetheless, it was hospital based and OBEs were studied with consideration to occupational characteristics. Second, reporting bias is possible because of possible underestimation of the response rate. Third this study might underestimate the true prevalence of HCV infections as some HCWs refused to participate perhaps because they suspected that they were already infected and did not want to know their status.

### Conclusion

This is a pilot Tunisian hospital based study done in order to determine the magnitude of hepatitis C infection and occupational exposures to blood and body fluids among HCWs. The actual infection rate was comparable to the general population, despite the high exposure to blood and body floods (56.7%). Doctors and participants working at surgical departments were more exposed than others categories. Our findings can help public health officers to develop preventive measures in order to monitor occupational exposure to HCV and others blood borne pathogens and to promote patient and HCWs safety. We recommend to preparing regular and up to date training sessions to increase the awareness about adherence to safety procedures among all HCWs.

## Supporting information

S1 File (SAV)

## Abbreviations

HCV: Hepatitis C Virus
HCWs: Health Care Workers
NSI: Needle stick injury
OBE: Occupational Blood or body fluid Exposure
FBUH: Fattouma Bourguiba University Hospital
IQR: Inter-Quartile range

## References

[1] Petruzziello A, Marigliano S, Loquercio G, Cozzolino A, Cacciapuoti C. Global epidemiology of hepatitis C virus infection: An up-date of the distribution and circulation of hepatitis C virus genotypes. World Journal of Gastroenterology. 2016; 22:7824–40. doi: 10.3748/wjg.v22.i34.7824 27678366 PMC5016383

[2] Global, regional, and national incidence, prevalence, and years lived with disability for 301 acute and chronic diseases and injuries in 188 countries, 1990–2013: a systematic analysis for the Global Burden of Disease Study 2013. Published 2015. Accessed 2020-25-02. Available from: https://www.ncbi.nlm.nih.gov/pmc/articles/PMC4561509/. 10.1016/S0140-6736(15)60692-4 PMC4561509 26063472

[3] Viral hepatitis and the Global Burden of Disease: a need to regroup. Journal of Viral Hepatitis. Published 2013. Accessed 2020-25-02. Available from: https://onlinelibrary.wiley.com/doi/abs/10.1111/jvh.12123. 10.1111/jvh.12123 23910643

[4] Centers for Disease Control and Prevention. Viral Hepatitis Surveillance—United States, 2017. Atlanta: US Department of Health and Human Services, Centers for Disease Control and Prevention; 2019. Published 2019. Accessed 2020-25-02. Available from: https://www.cdc.gov/hepatitis/statistics/2017surveillance/index.htm.

[5] Liang TJ, Rehermann B, Seeff LB, Hoofnagle JH. Pathogenesis, Natural History, Treatment, and Prevention of Hepatitis C. Ann Intern Med. 2000;132(4):296. doi: 10.7326/0003-4819-132-4-200002150-00008 10681285

[6] Garozzo A, Falzone L, Rapisarda V, Marconi A, Cinà D, Fenga C, et al. The risk of HCV infection among health-care workers and its association with extrahepatic manifestations. Molecular Medicine Reports. 2017;15:3336–9. 28339065 10.3892/mmr.2017.6378 PMC5428681

[7] Marković-Denić L, Branković M, Maksimović N, Jovanović B, Petrović I, Simić M, et al. Occupational exposures to blood and body fluids among health care workers at university hospitals. Serbian Archives of Medicine. 2013;141:789–93. doi: 10.2298/sarh1312789m 24502099

[8] Westermann C, Peters C, Lisiak B, Lamberti M, Nienhaus A. The prevalence of hepatitis C among healthcare workers: a systematic review and meta-analysis. Occupational and Environmental Medicine. 2015;72:880–8. doi: 10.1136/oemed-2015-102879 26438666 PMC4680146

[9] Varghese GM, Abraham OC, Mathai D. Post-exposure prophylaxis for blood borne viral infections in healthcare workers. Postgraduate Medical Journal. 2003;79:324–8. doi: 10.1136/pmj.79.932.324 12840120 PMC1742734

[10] Gillen M, McNary J, Lewis J, Davis M, Boyd A, Schuller M, et al. Sharps-Related Injuries in California Healthcare Facilities: Pilot Study Results From the Sharps Injury Surveillance Registry. Infection Control & Hospital Epidemiology. 2003;24:113–21. 12602693 10.1086/502181

[11] Egro FM, Nwaiwu CA, Smith S, Harper JD, Spiess AM. Seroconversion rates among health care workers exposed to hepatitis C virus–contaminated body fluids: The University of Pittsburgh 13-year experience. American Journal of Infection Control. 2017;45:1001–5. doi: 10.1016/j.ajic.2017.03.011 28449917

[12] Alhamoudi H, Alhalabi N, Zein M, Ibrahim N. Hepatitis C virus antibodies are absent among high risk group of health care workers in Damascus Hospital. Arab Journal of Gastroenterology. 2018;19:80–3. doi: 10.1016/j.ajg.2018.02.012 29934266

[13] Hebo HJ, Gemeda DH, Abdusemed KA. Hepatitis B and C Viral Infection: Prevalence, Knowledge, Attitude, Practice, and Occupational Exposure among Healthcare Workers of Jimma University Medical Center, Southwest Ethiopia. Scientific World Journal 2019. Published 2019. Accessed 2019-08-06. Available from: https://www.ncbi.nlm.nih.gov/pmc/articles/PMC6377947/. 10.1155/2019/9482607 PMC6377947 30853866

[14] Zaaijer HL, Appelman P, Frijstein G. Hepatitis C virus infection among transmission-prone medical personnel. Eur J Clin Microbiol Infect Dis. 2012;31:1473–7. doi: 10.1007/s10096-011-1466-9 22045049 PMC3364421

[15] Strickland GT, El-Kamary SS, Klenerman P, Nicosia A. Hepatitis C vaccine: supply and demand. Lancet Infect Dis. 2008;8:379–86. doi: 10.1016/S1473-3099(08)70126-9 18501853

[16] A special meeting review edition: Advances in the Treatment of Hepatitis C Virus Infection From EASL 2015. Journal of Gastroenterology and Hepatology. 2015;11:1–23. 26504459

[17] Kaabia N, Ben Jazia E, Hannachi N, Khalifa M, Dhouibi S, Dabbabi F, et al. Prévalence de l’hépatite virale C chez le personnel de santé au Centre tunisien. Médecine et Maladies Infectieuses. 2009;39:66–7. doi: 10.1016/j.medmal.2008.10.007 19041206

[18] Azzouzi Y EL Bakkali M, Khadmaoui A, Ahami AOT, Hamama S. Occupational exposure to blood among health-care workers: knowledge, attitude, practice and prevention of the Gharb region in Morocco. International Journal of Innovation and Applied Studies. 2014;7:557–70.

[19] Jahic R, Piljic D, Porobic-Jahic H, Custović A, Petrovic J, Piljic D. Epidemiological Characteristics of the Accidental Exposures to Blood-Borne Pathogens Among Workers in the Hospital. Medical Archives. 2018;72:187–91. doi: 10.5455/medarh.2018.72.187-191 30061764 PMC6021162

[20] Alemayehu T, Worku A, Assefa N. Sharp Injury and Exposure to Blood and Body Fluids among Health Care Workers in Health Care Centers of Eastern Ethiopia. International Journal of Occupational and Environmental Medicine. 2016;7:714–172‑80. doi: 10.15171/ijoem.2016.714 27393324 PMC6818079

[21] Beghdadli B, Ghomari O, Taleb M, Belhaj Z, Belabed A, del Kandouci AK, et al. Personnel at risk for occupational blood exposure in a university hospital in West Algeria. Sante Publique. 2009;21(3):253–261. 19863016

[22] Binull Peng, Tully PJ, Boss K, Hiller JE. Sharps injury and body fluid exposure among health care workers in an Australian tertiary hospital. Asia Pacific Journal of Public Health. 2008;20:139–47. doi: 10.1177/1010539507312235 19124307

[23] Myers DJ, Epling C, Dement J, Hunt D. Risk of sharp device-related blood and body fluid exposure in operating rooms. Infection Control & Hospital Epidemiology. 2009;29: 1139–48. 10.1086/592091 18991506

[24] Lee JH, Cho J, Kim YJ, Im SH, Jang ES, Kim J-W, et al. Occupational blood exposures in health care workers: incidence, characteristics, and transmission of bloodborne pathogens in South Korea. BMC Public Health. Published 2017. Accessed 2019-08-06. Available from: https://www.ncbi.nlm.nih.gov/pmc/articles/PMC5648449/. 10.1186/s12889-017-4844-0 PMC5648449 29047340

[25] Nissaf BA. Plan National d’élimination de l’Hépatite Virale C Tunisie 2016–2023. Published 2017. Accessed 2020-07-24. Available from:https://www.infectiologie.org.tn/pdf_ppt_docs/congres2017/infectionsvirales/c5PNE-VHC-medecinsreferents.pdf.

[26] Alqahtani JM, Abu-Eshy SA, Mahfouz AA, El-Mekki AA, Asaad AM. Seroprevalence of hepatitis B and C virus infections among health students and health care workers in the Najran region, southwestern Saudi Arabia: the need for national guidelines for health students. BMC Public Health. 2014;14:577. doi: 10.1186/1471-2458-14-577 24912684 PMC4059075

[27] Yazdanpanah Y, De Carli G, Migueres B, Lot F, Campins M, Colombo C, et al. Risk factors for hepatitis C virus transmission to health care workers after occupational exposure: A European case-control study. Clinical Infectious Diseases. 2005;41:1423–30. doi: 10.1086/497131 16231252

